# Flavonoids on diabetic nephropathy: advances and therapeutic opportunities

**DOI:** 10.1186/s13020-021-00485-4

**Published:** 2021-08-07

**Authors:** Qichao Hu, Caiyan Qu, Xiaolin Xiao, Wenwen Zhang, Yinxiao Jiang, Zhao Wu, Dan Song, Xi Peng, Xiao Ma, YanLing Zhao

**Affiliations:** 1 State Key Laboratory of Southwestern Chinese Medicine Resources, School of Pharmacy, Chengdu University of Traditional Chinese Medicine, Chengdu, 611137 China; 2 Hospital of Chengdu University of Traditional Chinese Medicine, School of Clinical Medicine, Chengdu University of Traditional Chinese Medicine, Chengdu, 611137 China; 3 Department of Pharmacy, The Fifth Medical Center of PLA General Hospital, Beijing, 100039 China

**Keywords:** Diabetic nephropathy, Flavonoids, Oxidative stress, Kidney fibrosis, Podocyte autophagy

## Abstract

With the advances in biomedical technologies, natural products have attracted substantial public attention in the area of drug discovery. Flavonoids are a class of active natural products with a wide range of pharmacological effects that are used for the treatment of several diseases, in particular chronic metabolic diseases. Diabetic nephropathy is a complication of diabetes with a particularly complicated pathological mechanism that affects at least 30% of diabetic patients and represents a great burden on public health. A large number of studies have shown that flavonoids can alleviate diabetic nephropathy. This review systematically summarizes the use of common flavonoids for the treatment of diabetic nephropathy. We found that flavonoids play a therapeutic role in diabetic nephropathy mainly by regulating oxidative stress and inflammation. Nrf-2/GSH, ROS production, HO-1, TGF-β1 and AGEs/RAGE are involved in the process of oxidative stress regulation. Quercetin, apigenin, baicalin, luteolin, hesperidin, genistein, proanthocyanidin and eriodictyol were found to be capable of alleviating oxidative stress related to the aforementioned factors. Regarding inflammatory responses, IL-1, IL-6β, TNF-α, SIRT1, NF-κB, and TGF-β1/smad are thought to be essential. Quercetin, kaempferol, myricetin, rutin, genistein, proanthocyanidin and eriodictyol were confirmed to influence the above targets. As a result, flavonoids promote podocyte autophagy and inhibit the overactivity of RAAS by suppressing the upstream oxidative stress and inflammatory pathways, ultimately alleviating DN. The above results indicate that flavonoids are promising drugs for the treatment of diabetic nephropathy. However, due to deficiencies in the effect of flavonoids on metabolic processes and their lack of structural stability in the body, further research is required to address these issues.

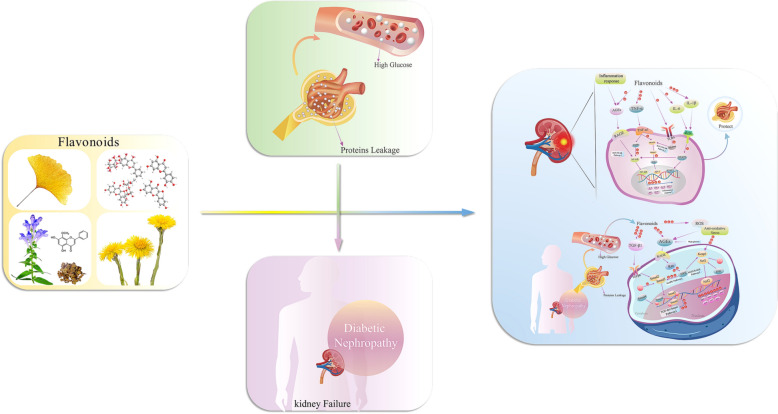

## Introduction

With the rapid development of high-throughput screening technology, natural products that possess a wide spectrum of bioactive effects have attracted public attention as potential new drugs [1]. Natural products with multiple pharmacological targets can be obtained from plants, animals, minerals or microorganisms, and the application of such products as Chinese herbal medicines can be traced back thousands of years [2, 3]. To date, many natural products have been advanced from experimental research to clinical application due to the continuous improvements in chemical technologies and biological methods [4]. For instance, polysaccharide components derived from *Poria cocos* (Schw.) Wolf. have been confirmed to have antitumour activity. Artemisinin extracted from *Artemisia annua* can be used to treat malaria. In general, natural products with multiple bioactive effects are good candidates to explore and evaluate in the area of drug discovery [5, 6].

Diabetes mellitus is a group of metabolic diseases characterized by hyperglycaemia that is divided into type I and type II. Recent studies have shown that long-term high glucose levels cause chronic damage to and dysfunction of several tissues, in particular the eyes, kidneys, heart, blood vessels, and nerves [7, 8]. Diabetic nephropathy (DN) is the most common complication of diabetes and affects at least 30% of diabetic patients, representing great burden on public health [9]. Studies have shown that the prevalence of chronic kidney disease (CKD) in China has reached 13.4%; the incidence of CKD is increasing annually, and it has become a serious public health concern [10]. As a typical progressive CKD, DN involves an intricate pathological mechanism accompanied by hyperglycaemia, excessive levels of reactive oxygen species (ROS) and impaired podocyte autophagy. In addition, DN is a key risk factor for a variety of adverse prognostic outcomes as it directly affects the cardiovascular system, especially in patients with diabetic nephropathy [11, 12]. As several factors are affected involving complex pathological mechanisms, effective, specific drugs for the treatment of DN are currently scarce. Thus, it is critical to find agents that can be used to effectively treat DN. To date, natural products have been shown to have a significant effect on inhibiting the development of DN. For example, salidroside from *Rhodiola rosea* L. has been reported to alleviate DN by downregulating the TGF-β1/Smad2/3 pathway [13]. Qi and his colleagues found that chromium picolinate could reverse DN by inhibiting oxidation and inflammatory pathways, and it is expected to be approved for use as a complementary medicine [14]. Additionally, cyanidin-3-glucoside from black rice has been confirmed to alleviate DN by downregulating TGF-β1/Smad2/3 pathway-associated extracellular matrix aggregation [15, 16].

Among several natural compounds, flavonoids have outstanding pharmacological effects, including antidiabetic, anti-inflammatory, antioxidative stress and antihypertension effects [17, 18]. In particular, flavonoids used in traditional Chinese medicines have demonstrated significant advantages for the treatment of DN. For example, baicalin has been confirmed to reverse renal damage and efficiently prevent the progression of CKD in streptozotocin-induced DN mice. Kaempferol has been shown to reduce kidney damage via its antioxidant and anti-inflammatory effects.

## Search strategies

For this narrative review, research articles on the treatment of diabetic nephropathy with flavonoids were collected from PubMed, the Cochrane Library Web of Science, and the EMBASE database. We have systematically explained the pharmacological mechanism of flavonoids in diabetic nephropathy, providing a reference for future research. The following types of article were excluded from our review: studies lacking scientific value and those with obvious methodological errors. Overall, we discuss articles regarding a total of 15 flavonoids used for the treatment of DN.

## Diabetic nephropathy

The pathogenesis of DN is complex and remains unclear, but research to date has shown that renal artery hypertension caused by hyperglycaemia, excessive levels of reactive oxygen species (ROS) and impaired podocyte autophagy are closely related to the occurrence of DN.

**Fig. 1:**
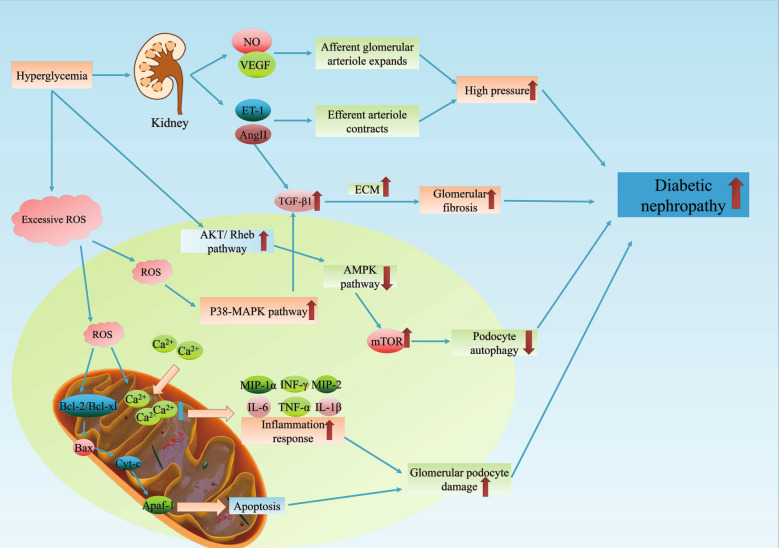
Pathogenesis of diabetic nephropathy. The hyperglycaemia state prompts the kidney to release VEGF and NO to expand afferent glomerular arterioles and AngII and ET-1 to contract efferent arterioles, which causes high blood pressure and leads to the occurrence of DN. TGF-β upregulation by Ang II promotes the process of glomerular fibrosis and aggravates DN. In hyperglycaemia, excessive ROS accumulate and open the mPTP, causing the intracellular Ca^2+^ concentration to increase, activating several apoptotic enzymes and inflammatory mediators and inducing glomerular podocyte damage. ROS can also activate the P38-MAPK pathway to increase the synthesis of TGF-β, which increases the level of ECM proliferation, promoting inflammation and fibrosis of the kidneys and ultimately leading to the occurrence of DN. High glucose can activate the AKT and Rheb pathways, inhibit the AMPK pathway, and promote the activation of mTOR to inhibit podocyte autophagy and promote the progression of DN

A hyperglycaemic state prompts the kidneys to release vascular endothelial growth factor (VEGF) and NO to expand afferent glomerular arterioles and release angiotensin II (AngII) and endothelin-1 (ET-1) to contract efferent arterioles, which leads to high blood pressure and the occurrence of DN [19]. Likewise, TGF-β upregulation by Ang II promotes glomerular fibrosis and aggravates DN [20]. On the other hand, in a state of hyperglycaemia, the human body accumulates excessive ROS, opening the mitochondrial permeability transition pores (mPTP) and causing the intracellular Ca^2+^ concentration to increase; subsequently, a series of apoptotic enzymes and several inflammatory mediators are activated, causing glomerular podocyte damage [21]. In addition, ROS can also activate the P38-MAPK pathway to increase the synthesis of TGF-β, which increases the level of extracellular matrix (ECM) proliferation. The above changes can promote inflammation and fibrosis of the kidneys and ultimately lead to the occurrence of DN [22]. In addition, podocyte autophagy is considered to be a key process that protects kidney cells, and impaired podocyte autophagy aggravates kidney cell damage. High glucose levels can activate the AKT and Rheb pathways, inhibit the AMPK pathway, and subsequently promote the activation of mTOR to inhibit podocyte autophagy and promote the progression of DN [23] (Fig. 1).

Due to the complex pathogenesis of diabetic nephropathy, there is currently a lack of clinically effective therapeutic drugs [24, 25]. Currently, the clinical treatment of DN mainly involves hypoglycaemic drugs, a combination of hypotensive drugs and hypolipidaemic drugs. Studies have shown that intensive blood sugar control can slow down the occurrence and development of diabetic microvascular complications. Angiotensin-converting enzyme inhibitors (ACEIs) or angiotensin receptor antagonists (ARBs) are the drugs of choice for patients with diabetic nephropathy [26, 27]. In addition, blood lipids are closely related to blood sugar levels, and most patients with type 2 diabetes have concurrent dyslipidaemia. Dyslipidaemia increases the burden on the kidneys of patients with DN and accelerates the process of DN. DN may also cause dyslipidaemia. Based on the above factors, it is necessary to develop a specific drug for the treatment of DN.

## Flavonoids for the treatment of diabetic nephropathy

### Quercetin

**Fig. 2:**
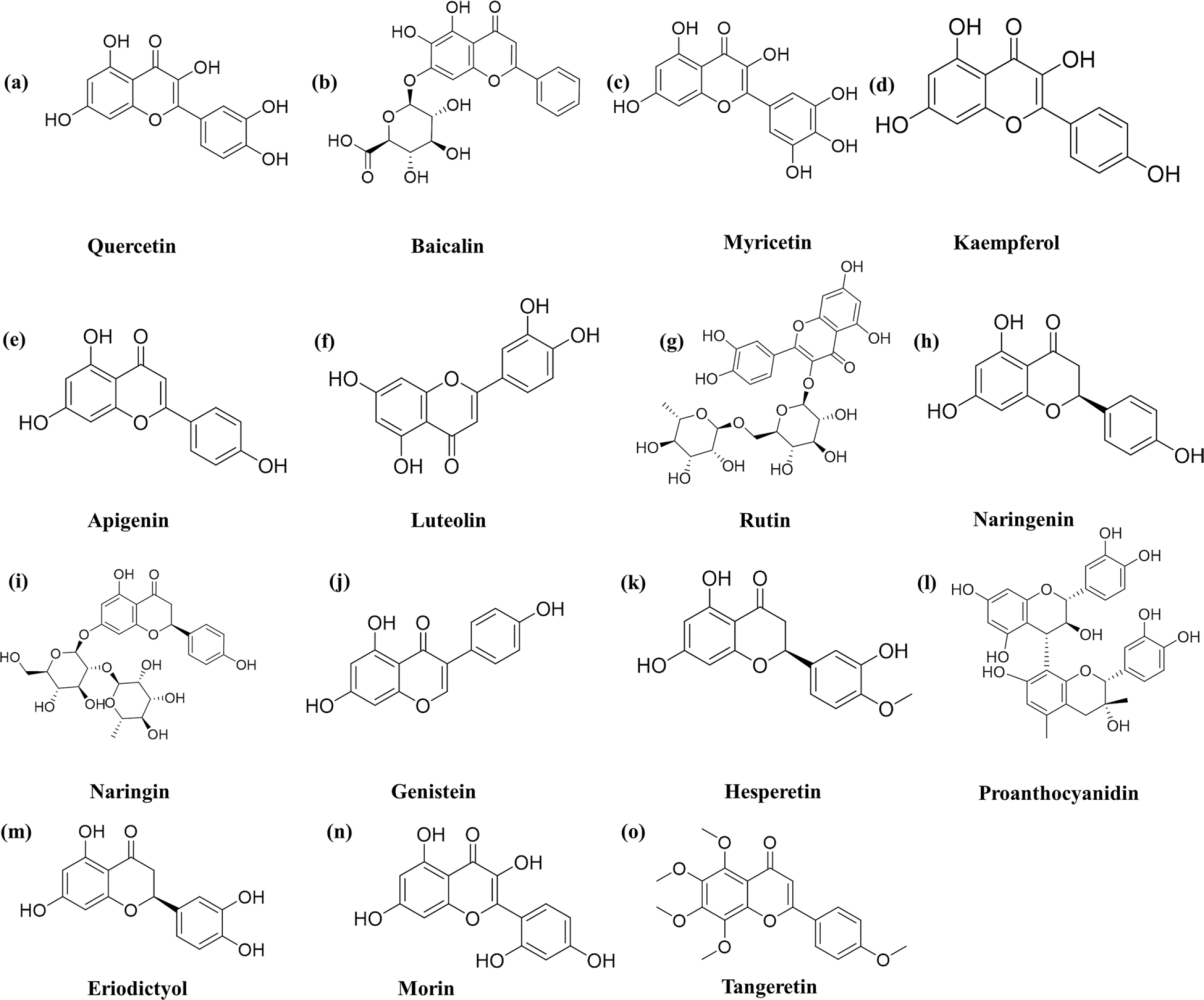
Chemical structures of the active flavonoids discussed in this review

Quercetin, rich in the stems and leaves of buckwheat, sea buckthorn, hawthorn, and onion, exists mostly in the form of glycosides, such as rutin and hyperoside, and can be extracted by alkaline extraction and acid precipitation [28, 29]. The formula of quercetin is shown in Fig. 2a. Glycosylation and methylation of quercetin are two instrumental modification strategies that have been used for several years to significantly improve its bioavailability [30]. Studies to date have shown that quercetin is beneficial for the treatment of several metabolic diseases. Evidence has shown that quercetin plays a significant role in reversing of diabetic nephropathy by reducing oxidative stress, fighting inflammation and eliminating free radicals.

Notably, quercetin is a free radical scavenger and superoxide radical inhibitor, indicating that it has strong antioxidant properties. A study by Elbe revealed that quercetin at 25 mg/kg can prevent streptozotocin-induced oxidative stress in diabetic rats by lowering lipid peroxidation and increasing superoxide dismutase (SOD) and catalase (CAT) activity to decrease the rats’ weight [31]. Moreover, Iskende administered 100 mg/kg quercetin to streptozotocin-induced diabetic rats, and the results showed that oxidative stress and NF-κB levels increased while SIRT1 levels decreased. Additionally, Iskende proposed that oxidative stress is an important criterion for the detection of potential complications in experimentally induced diabetes [32]. On the other hand, Anjaneyulu administered quercetin at a dose of 10 mg/kg orally to streptozotocin-induced diabetic rats for 4 weeks, and the results strongly suggested that oxidative stress plays a critical role in the pathophysiology of diabetic nephropathy, and that quercetin as a dietary supplement may act as an antioxidant and prevent or treat diabetic complications. Using the same model, Wang and his colleagues concluded that quercetin at 25 ~ 100 mg/kg inhibits the activation of the renal NLRP3 inflammasome, thereby alleviating the associated nephrotoxicity in streptozotocin-induced diabetic rats [34]. Similar to Wang, Gomes and colleagues also studied the mechanism of quercetin from the perspective of kidney disease protection, and their outcomes revealed that chronic oral treatment with 10 mg/kg quercetin can reduce oxidative stress and apoptosis in streptozotocin-induced DN mice as a result of decreased ROS levels [35]. Tang found that 50 mg/kg/d quercetin liposomes effectively alleviate streptozotocin-induced diabetic nephropathy in diabetic rats via its antioxidant capacity and ability to scavenge free radicals; additionally, they showed that the levels of MDA, TNF-α, IL-1β, and AGEs decreased and the activities of SOD and GSH-Px increased [36]. Lei found that 50 mg/kg quercetin can significantly inhibit the overexpression of TGF-β1 and connective tissue growth factor (CTGF) in streptozotocin-induced diabetic rats induced and that elevated levels of urine albumin excretion (UAE), serum creatinine (sCr), blood urea nitrogen (BUN), and creatinine clearance (Ccr) were also significantly alleviated [37]. Furthermore, Wang treated type 2 diabetic mice orally with three 150 mg/kg ethanol extracts from green cocoons, that are rich in quercetin, for 7 weeks. The results demonstrated that quercetin may have antifibrosis and anti-inflammatory activities that inhibit the regulation of the TNF-α-p38 MAP kinase signalling pathway, reducing blood sugar levels and improving the body weight of the type 2 diabetic animals [38]. Jiang administered 5–100 mg/kg quercetin to Leprdb/Leprdb(db/db) mice and found that quercetin not only significantly downregulated the expression of low-density lipoprotein (LDLr), HMGCR, SREBP-2 and SCAP but also reduced the changes in the renal lipid profile and lipid droplet accumulation in the mice. He hypothesized that this effect may be related to the improvement of lipid metabolism resulting from the effect of quercetin on the SCAP-SREBP2-LDLr signalling pathway [39]. In addition, Du found that 100 ~ 150 mg/kg quercetin played an antiproliferative role by reactivating the Hippo signalling pathway, thus inducing the proliferation of mesangial cells and reduction of kidney fibrosis in Leprdb/Leprdb(db/db) mice, leading to improved renal function and blood lipid levels [40]. In a different study, Tong administered a single abdominal subcutaneous injection of quercetin to Sprague–Dawley rats at a dose of 10 mg/kg and collected blood and left kidneys for analysis. According to his results, quercetin can reduce oxidative stress-related kidney damage, limit inflammatory cell infiltration into the kidney, and downregulate intercellular adhesion molecular-1 (ICAM-1) expression [41]. Chen and colleagues reported that quercetin at 100 µM reversed the effects of high glucose (HG) on human mesangial cells (HMCs), resulting in the upregulation of NF-κB and MCP-1 expression. Therefore, they concluded that the effect of quercetin is partly mediated by the NF-κB signalling pathway [42] (Table 1).

**Table 1:** Information on flavonoids for the treatment of diabetic nephropathy

Compound	Animal/Cell model	Dosage	Target/Pathways/Mechanism	Reference
Quercetin	Streptozotocin-induced DN rats	50 mg/kg/d	TNF-α/IL-1β/AGEs	[31]
	Streptozotocin-induced DN rats	150–350 mg/kg	TNF-α-p38 MAP kinase signalling pathway	[32]
	Leprdb/Leprdb(db/db) mice	50–100 mg/kg	SCAP-SREBP2-LDLr signalling pathway	[33]
	Leprdb/Leprdb(db/db) mice	100–150 mg/kg	Hippo pathway	[40]
	Sprague Dawley rats	10 mg/kg	IcaM-1	[35]
	Streptozotocin-induced DN rats	100 mg/kg	NF-kB/SIRT1	[36]
	Streptozotocin-induced DN rats/NRK-52E cells	10 mg/kg	reduce ROS	[39]
	Streptozotocin-induced DN rats	25 mg/kg	TGF-β1	[38]
	Streptozotocin-induced DN rats	25–100 mg/kg	NLRP3	[34]
	Streptozotocin-induced DN rats	50 mg/kg	TGF-β1/CTGF	[37]
	Streptozotocin-induced DN rats	10 mg/kg	PKC/MARK pathway	[41]
	Human mesangial cell (HMC)	100 µM (in vitro)	NF-κB signalling pathway	[42]
Baicalin	High glucose-induced podocyte	6.25–25 μM *(*in vitro)	Sirtuin 1/NF-κB signalling pathway	[46]
	Streptozotocin-induced DN mice	15–45 mg/kg/d	microRNA-124/TLR4/NF-κB axis	[47]
	Streptozotocin-induced DN mice	160 mg/kg	MAPK pathway/NF-κB signalling// TGF-β/Smad3 pathway	[48]
Kaempferol	Streptozotocin-induced DN mice	10 mg/kg	TRAF6	[51]
	Streptozotocin-induced DN mice/GLUTag cell lines	50–200 mg/kg;1–50 μM (in vitro)	GLP-1/RhoA/Rho kinase	[53]
	NRK-52E and RPTEC cells	5–50 μM*(*in vitro)	RhoA/Rho kinase	[52]
Myricetin	Streptozotocin with cadmium induced DN rats	1.0–1.5 mg/kg	SREBP-1a/SREBP-1c/SREBP-2/TGF-β1/VEGF/PPAR-α	[59]
	Streptozotocin with cadmium induced DN rats	1.0 mg/kg	GLUT-2/GLUT-4/IRS-1/IRS-2/PKB	[58]
	Streptozotocin-induced DN rats	6 mg/d	GPx/XO	[57]
Rutin	Streptozotocin-induced DN rats	100 mg/kg	MMPs	[63]
	Streptozotocin-induced DN rats	10–90 mg/kg	TGF-β1/Smad/ECM and TGF-β1/CTGF/ECM signalling pathways	[64]
	HRGECs	12.5–50 μM (in vitro)	ROS/Rhoa/ROCK Signalling Pathway	[65]
	Glomerular mesangial cells	0.2–0.8 μM (in vitro)	ACTA2 and p38 protein	[66]
	Alloxan-induced DN rats	100 mg/kg	AQP2/AQP3/V2R	[67]
	Alloxan-induced DN rats	100 mg/kg	GF-β1/GRP78/CHOP	[68]
Apigenin	Streptozotocin-induced DN mice	20 mg/kg	TNF-α/IL-6/NF-κB/MAPK signalling pathway	[72]
	HK-2 cells	100–200 μM (in vitro)	Nrf2/HO-1	[71]
	Streptozotocin-induced DN mice	25–50 mg/kg	Nrf2/HO-1/NF-kB Signalling Pathway	[71]
Luteolin	Streptozotocin-induced DN rats	80 mg/kg	Nphs2	[74]
	Leprdb/Leprdb(db/db) mice	50 mg/kg	STAT3 pathway	[75]
	Nesangial cells MPC-5 cells	30 μM (in vitro)	IL-1β/NLRP3	[76]
	Streptozotocin-induced DN rats	200 mg/kg	SOD/MDA/HO-1	[77]
Naringin	Rat glomerular mesangial cells	5–80 μM (in vitro)	NLRP3	[80]
	Streptozotocin-induced DN rats	20–80 mg/kg	NOX4	[81]
Naringenin	Streptozotocin-induced DN rats	5–10 mg/kg	IL-1	[82]
	Streptozotocin-induced DN rats	50 mg/kg	let-7a/TGFBR1 signalling pathway	[83]
	Streptozotocin-induced DN rats/NRK-52E cells	25–75 mg/kg; 0.01–1 μM (in vitro)	CYP4A/20-HETE/PPARs	[84]
Hesperidin	Streptozotocin-induced DN rats	50–150 mg/kg	Nrf2/ARE/glo1 pathway	[88]
	Streptozotocin-induced DN rats	100 mg/kg	α-KL/FGF-23 pathway	[89]
	Streptozotocin-induced DN rats	40 mg/kg	TGF-β1-ILK-Akt signalling pathway	[90]
Genistein	Streptozotocin-induced DN rats	10 mg/kg	ERK	[93]
	Alloxan-induced DN rats	0.025–0.1%	NF-kB/(MCP-1)/TGFβ-1	[94]
	Mouse podocyte cell lines	20 μM (in vitro)	mTOR signalling pathway	[95]
Proanthocyanidin	Streptozotocin-induced DN rats	250 mg/kg	Nrf2 signalling pathway	[98]
	Streptozotocin-induced DN rats	250 mg/kg	Caspase-12 pathway	[99]
	Streptozotocin-induced DN rats	250 mg/kg	AGEs/RAGE	[100]
	Streptozotocin-induced DN rats	125–500 mg/kg	AMPK-SIRT1-PGC-1a signalling/PGC-1α/SIRT1/AMPK	[101]
	Streptozotocin-induced DN rats	500 mg/kg	TGF-β1/AGEs/RAGE/CTGF	[102]
Eriodictyol	Mesangial cells MPC-5 cells	0–25 μm (in vitro)	Akt/NF‐κB pathway	[104]
Morin	Mesangial cells MPC-5 cells	25–50 μm (in vitro)	p38 MAPK/JNK signalling pathway	[107]
Tangeretin	db/db mice	10 mg/kg	EMT/E-cadherin/P-cadherin	[109]
	Human glomerular mesangial cells	0–26 μm (in vitro)	ROS/MDA/FN/ERK signalling pathway	[110]

### Baicalin

Baicalin, a key flavonoid compound, is mainly obtained from *Scutellaria baicalensis* Georgi by alkaline extraction and acid precipitation. Baicalin has obvious pharmacokinetic characteristics, including gastrointestinal hydrolysis, enterohepatic circulation, carrier-mediated transport and complex metabolism. Owing to its low bioavailability, baicalin is mainly administered in the form of nanoparticles and liposomes. Its formula is shown in Fig. 2b [43]. In recent years, several studies have shown that the effects of baicalin for the treatment of metabolic diseases are significant; baicalin is especially effective for the treatment of diabetes complications [44, 45].

Li investigated the reversal effect of baicalin on podocyte apoptosis induced by high glucose to discover the mechanism by which baicalin alleviates DN. The results indicated that baicalin can relieve DN by increasing the expression level of sirtuin 1 in high glucose-induced podocytes and inhibiting the NF-κB pathway [46]. Zhang and colleagues reported that baicalin at doses of 15–45 mg/kg/d reversed renal damage and efficiently prevented the progression of CKD in streptozotocin-induced DN mice. Furthermore, they found that baicalin prevents renal fibrosis in DN mice by increasing miR-124 levels and blocking the downstream TLR4/NF-κB pathway [47]. Using the same model, Zheng revealed that 160 mg/kg baicalin administration significantly alleviated kidney damage, metabolic disorders and renal fibrosis by anti-inflammatory and anti-oxidative stress mechanisms. Zheng’s results suggested that baicalin inhibits inflammation through the NF-κB signalling pathway, blocks extracellular matrix accumulation through the TGF-β/Smad3 pathway, and regulates cell proliferation via the insulin-like growth factor (IGF)-1/IGF-1 receptor/p38 mitogen-activated protein kinase (MAPK) pathway [48].

### Kaempferol

Kaempferol, an active flavonoid compound widely found in tea, cruciferous vegetables, and several fruits, is generally extracted by high-performance liquid chromatography and been proven in recent years to exert several pharmacological effects, including anti-inflammatory, anti-oxidative stress, and antiatherosclerotic effects [49, 50]. Its formula is shown in Fig. 2d.

Luo and colleagues explored the association between the attenuating effect of kaempferol in streptozotocin-induced DN mice and tumour necrosis factor receptor associated factor 6 (TRAF6). They found that kaempferol at a dose of 10 mg/kg significantly impaired kidney damage due to its antioxidant and anti-inflammatory effects, which correlated with a decrease in TRAF6 levels [51]. A recent study by Sharma indicated that kaempferol can attenuate DN by inhibiting the inflammatory signal mediated by RhoA/Rho kinase, which has been confirmed to have a preventive effect on several enzymes that exert antioxidant and cytoprotective effects. The results suggested that 5–50 μM kaempferol inhibits the activity of RhoA/Rho kinase and decreases the expression of oxidative stress and its related proinflammatory factors (TNF-α and IL-1β) in NRK-52E and RPTEC cells [52]. Interestingly, based on the finding that kaempferol potentially targets RhoA/Rho kinase, Sharma and colleagues subsequently conducted a comprehensive in vitro and in vivo combination experiment to explore the mechanism of kaempferol treatment for DN. The results showed that kaempferol can improve kidney injury and reduce fibrosis by enhancing the release of GLP-1 and insulin and inhibiting RhoA/Rho kinase [53].

### Myricetin

Myricetin is commonly found in tea and wine produced from berries, vegetables, and several plants. Myricetin exists in a free form or forms structures with other compounds via glycosidic bonds, for example, myricetin-3-*O*-(3″-acetyl)-α-L-arabinosid, myricetin-3-O-(4″-acetyl) -α-l-arabinosid, and myricetin-3-*O*-α-l-rhamnoside [54]. Its formula is shown in Fig. 2c. Studies have shown that myricetin exerts a wide range of pharmacological effects, including anticancer, anti-inflammatory, antioxidant and antiviral effects [55, 56]. Accumulating experimental data have shown that myricetin plays an essential therapeutic role in the attenuation of hyperglycaemia and hyperlipidaemia. Studies have shown that the progression of DN is closely related to abnormal lipid metabolism in patients, which provides theoretical support for the use of myricetin as a treatment for DN.

Ozcan demonstrated that myricetin considerably reduces the blood sugar level of streptozotocin-induced DN rats and improves the activity of glutathione peroxidase (GPx) and xanthine oxidase (XO) in kidney tissue, indicating that myricetin is a potential therapeutic agent for DN [57]. Kandasamy and colleagues revealed the protective effects of myricetin in streptozotocin and cadmium-induced DN rats. They found that 1.0 mg/kg myricetin alleviated the progression of DN and improved kidney function by increasing the expression of insulin, glycogen, glycogen synthase and insulin signalling molecules, including glucose transporter-2 (GLUT-2), glucose transporter-4 (GLUT-4), insulin receptor-1 (IRS-1), insulin receptor-2 (IRS-2) and protein kinase B (PKB) [58]. Furthermore, Kandasamy et al. investigated the mechanism of the lipid metabolism regulation of myricetin on streptozotocin in cadmium-induced DN rats. The results suggested that myricetin at doses of 1.0–1.5 mg/kg upregulated sterol regulatory element binding protein-1a (SREBP-1a), SREBP-1c, SREBP-2, TGF-β1, VEGF and peroxidase PPAR-α (PPAR-α) expression leading to the amelioration of abnormal lipid and glucose metabolism, thereby alleviating the progression of renal fibrosis [59].

### Rutin

Rutin (3,3′,4′,5,7-pentahydroxyflavone-3-rhamnoside, Fig. 2g) is a flavonoid that is abundant in tea and plants such as passionflower, buckwheat, and apple[60]. Rutin is a glycoside comprising quercetin and disaccharide rutose that has recently been reported to be an ROS scavenger that can promote insulin secretion [61, 62]. The Stanely Mainzen Prince group revealed that 100 mg/kg rutin suppresses the progression of DN by protecting the activity of matrix metalloproteinases (MMPs) and reducing plasma glucose levels in streptozotocin-induced DN rats [63].

Based on the results of the study of the Prince group, Hao and colleagues continued to explore the mechanism of the protective effect of rutin in an early experimental DN model. They found that 10–90 mg/kg rutin administration significantly reduced the levels of creatinine, blood urea nitrogen (BUN) and blood sugar by inhibiting the expression of AGEs, type IV collagen and laminin, TGF-β1, p-Smad 2/3 and CTGF, thereby inhibiting the progression of DN in streptozotocin-induced DN rats [64]. In addition, Wang et al. found that 12.5–50 μM rutin inhibited the RhoA/ROCK signalling pathway by reducing reactive oxygen species, thereby significantly preventing hyperglycaemia-induced destruction of renal endothelial barrier function in HRGECs, indicating that anti-oxidative stress effects are the mechanism by which rutin alleviates DN [65]. Furthermore, Han et al. found that at concentrations of 0.2, 0.4 and 0.8 mol/l, rutin obviously suppressed renal fibrosis in high glucose-induced glomerular mesangial cells by inhibiting human mesangial cell viability, ATP content, the expression of ACTA2 and p38 and improving the cell cycle progression of mesangial cells [66]. Additionally, in a model study of alloxan-induced DN experimental rats, Ganesan explored the alleviating effect of rutin on metabolic acidosis and renal fibrosis. The results suggested that 100 mg/kg rutin significantly reduced the levels of serum nitrogen compounds and metabolic acidosis-related genes (AQP2, AQP3 and V2R), thereby correcting metabolic acidosis in alloxan-induced DN rats and inhibiting the process of renal fibrosis [67].

In addition, the combination of rutin and antihypertensive drugs could be an effective way to treat DN. Ganesan investigated the therapeutic mechanism of the combination of rutin and ramipril to alleviate alloxan-induced DN in rats. They found that the combination of 100 mg/kg rutin and 5 mg/kg ramipril might alleviate DN by downregulating the activity of multiple TGF-β1-associated stress pathways and the expression of endoplasmic reticulum stress markers GRP78 and CHOP [68]. The results of the aforementioned study indicate that combination therapy with angiotensin-converting enzyme inhibitors and antioxidants could be a promising treatment strategy for DN.

### Apigenin

Apigenin is a common flavonoid compound that is rich in fruits and vegetables (onions, oranges and parsley) and exerts a wide range of pharmacological effects, such as anti-inflammatory, antioxidant, and anticancer effects. Its formula is shown in Fig. 2e. The results of recent studies have proven that apigenin could play a crucial role in diabetes therapy, and it is considered a promising agent for treating several diabetic complications [69, 70].

Malik and colleagues explored the underlying mechanisms of apigenin in DN via anti-inflammatory and antioxidant pathways. They found that 20 mg/kg apigenin treatment alleviated renal dysfunction, oxidative stress and renal fibrosis by inhibiting TGF-β1, fibronectin, and type IV collagen. Moreover, apigenin significantly suppressed the activation of the downstream inflammatory MAPK pathway by reducing the expression of TNF-α, IL-6 and NF-κB and reducing the expression of the apoptotic proteins Bax and caspase-3 [71]. Zhang revealed the therapeutic effects and potential mechanisms of apigenin on renal tubular epithelial cells exposed to hyperglycaemia. They found that 100–200 μM apigenin obviously reduced the apoptosis of renal tubular epithelial cells by inhibiting oxidative stress and increasing the expression of NF-E2-related factor 2 (Nrf2) and haem oxygenase-1 (HO-1) in a DN model using the high glucose-induced human renal tubular epithelial cell line HK-2 [71]. Furthermore, Li et al. used apigenin-loaded solid lipid nanoparticles (SLNPs) to explore the mechanisms of apigenin in DN through analyzing anti-oxidative stress pathways. The results showed that 25–50 mg/kg apigenin-SLNP treatment increased the expression of Nrf2 and HO-1 and inhibited the expression of NF-κB in streptozotocin-induced DN mice [72].

### Luteolin

Luteolin (3,4,5,7-tetrahydroxy flavone, Fig. 2f) is a natural flavonoid compound that exerts several pharmacological effects, including anti-inflammatory, anti-allergic and uric acid-lowering effects. Luteolin can be extracted from honeysuckle, chrysanthemum, nepeta and other plants as well as carrots, celery, sweet peppers, peppers, groundnuts and other vegetables and fruits [72, 73].

The results of recent studies have proven that luteolin exerts anti-inflammatory and antioxidant activities, indicating that it could be a novel treatment agent for kidney protection in DN patients. Xiong recently found that luteolin at a dose of 80 mg/kg protects the filtration function of the basement membrane by upregulating Nphs2 protein expression, which strikingly suppressed the apoptosis, deletion and fusion of streptozotocin-induced diabetic rat podocytes in high-glucose conditions. In addition, Xiong proposed that luteolin might inhibit glomerulosclerosis and maintain the relatively normal physiological structure of glomeruli, preventing the rapid DN-induced deterioration of the kidneys [74]. Zhang investigated the effects of luteolin on appropriated tubular injury in the kidneys of mice to explore the mechanism by which luteolin protects the kidneys in DN. The results showed that 50 mg/kg luteolin inhibited the anti-inflammatory response and oxidative stress by suppressing the activity of the STAT3 pathway, thus reducing renal fibrosis and delaying the progression of DN [75]. In an in vitro model, Yu and colleagues treated high-glucose exposed MPC-5 cells with luteolin to investigate the effect of luteolin on DN. The results suggested that luteolin significantly reduces the formation of pyrin domain-containing protein 3 (NLRP3) inflammasomes in HG-induced MPC-5 cells and the subsequent secretion of interleukin-1β (IL-1β), protecting podocytes from mercury-induced apoptosis and mitochondrial membrane potential collapse. On the other hand, the antiapoptotic effect of NLRP3 mainly occurs via the NLRP3 inflammasome [76]. Furthermore, Wang recently reported that 200 mg/kg luteolin increased SOD activity, reduced malondialdehyde (MDA) content and increased haemoxygenase-1 (HO-1) protein expression levels, which suggests that luteolin can prevent the morphological destruction of the kidneys in DN and improve the kidney redox balance [77].

### Naringenin and naringin

Naringenin forms glycosides with neohesperidoside at the 7-carbon position to produce the important metabolite, naringin. Naringin (Fig. 2i) and naringenin (Fig. 2h) are both crucial bioflavonoids derived from grapefruits and citrus fruits and have been reported to have anti-inflammatory, anti-apoptotic, anticancer and cardioprotective effects [78, 79].

Chen demonstrated that 5–80 μM naringin significantly inhibited the proliferation of high glucose-induced rat mesangial cells by downregulating the expression of the pyrin domain-containing-3 (NLRP3) inflammasome, which is correlated with inflammatory factors such as IL-1β and IL-18 [80]. In addition, Zhang and colleagues explored the potential mechanisms of naringin in streptozotocin-induced DN rats. The results suggested that naringin obviously reduced the renal damage induced by streptozotocin. In another study, naringin was shown to alleviate DN by downregulating the expression level of NADPH oxidase 4 (NOX4) [81].

Additionally, a recent study by Roy on the protective effects of naringenin on ND revealed that 5 mg/kg and 10 mg/kg naringenin significantly reversed renal impairment by downregulating the expression of inflammatory factor IL-1 and reducing the extent of oxidative stress in streptozotocin-induced DN rats [82]. Moreover, Yan et al. found that naringenin at a dose of 50 mg/kg affected the expression of Col4 and FN by upregulating the expression of let-7a in matrix metalloproteinases and further inhibiting the activity of the downstream TGF-β1/smad signalling pathway, which indicates that naringenin ameliorates renal impairment in streptozotocin-induced DN rats via the let-7a/TGFBR1 signalling pathway [83]. In addition, Ding investigated the protective effect of 25 and 75 mg/kg naringenin on DN, and the results suggested that naringenin increased the expression of CYP4A and the level of 20-hydroxyeicosatetraenoic acid (20-HETE), and simultaneously promoted the expression of PPARs. Further in vitro experiments also confirmed that naringenin (0.01, 0.1, 1 μmol/L) upregulated the expression of CYP4A, 20-HETE and PPARs in NRK-52E cells in a dose-dependent manner [84].

### Hesperetin

Hesperitin (Fig. 2k) is a bioflavonoid found in a wide range of citrus fruits that exerts several pharmacological effects, such as reducing the fragility of capillaries, protecting capillaries, and preventing capillaries from rupturing and bleeding [85, 86]. In the human body, hesperidin is deglycosylated via hesperetin-7-*O*-glucoside by two specific monoglycosidases, α-rhamnosidase and β-glucosidase and by one-step deglycosylation by α-rhamnosidase. Alkylation and deglycosylation yield hesperetin, which has pharmacological effects [87]. To date, hesperidin administration has proven beneficial for ameliorating diabetes, especially the complications of diabetes.

Chen and colleagues evaluated the protective effect of hesperetin on streptozotocin-induced diabetic rat kidneys and explored its underlying mechanism through the Nrf2/ARE/glo1 pathway. They found that 50 and 150 mg/kg hesperetin significantly upregulated the level of glyoxalase 1 (Glo-1), blocked the AGE/RAGE axis and suppressed related inflammation. Meanwhile, hesperidin administration increased the levels of Nrf2 and p-Nrf2 and upregulated the expression of the Nrf2/ARE signal transduction target gene γ-glutamyl half cystine synthase by activating the Nrf2/ARE pathway, resulting in the alleviation of DN [88]. Likewise, Eda noted that hesperidin at a dose of 100 mg/kg had antioxidant effects on streptozotocin-induced diabetic rats. They found that hesperidin reversed α-KL/FGF-23 axis disorders, increased α-KL levels in the serum, liver and kidney, and reduced FGF-23 and MDA expression levels. Accordingly, Eda suggested that the α-KL/FGF-23 signalling pathway might be a potential biological indicator of systemic toxicity and pathology associated with hesperidin treatment [89]. In addition, Zhang found that 15–80 mg/kg hesperetin obviously suppressed DN by inhibiting the TGF-β1-ILK-Akt signalling pathway in streptozotocin-induced diabetic rats. Zhang’s results indicated that the expression of TGF-β1 and its downstream effectors integrin-linked kinase (ILK) and Akt were inhibited [90].

### Genistein

Genistein (Fig. 2j), a common isoflavone component derived from soybean plants, exerts several pharmacological activities, such as inhibiting inflammation, promoting cell apoptosis, and regulating steroid hormone receptors and related metabolic pathways [91, 92]. Recently, evidence has suggested that genistein could play a crucial role in DN alleviation.

Elmarakby focused on the potential mechanism underlying the protective effect of tyrosine kinase inhibition against diabetes-induced organ damage. They found that 10 mg/kg genistein enhanced the beneficial effect of tyrosine kinase inhibitors on DN by increasing the expression of renal phosphotyrosine and the ratio of renal phospho-ERK/ERK [93]. In addition, Kim et al. found that 0.025%-0.1% genistein supplementation obviously prevented DN by inactivating the NF-kB and monocyte chemoattractant protein-1 (MCP-1) pathways as well as downregulating the expression of fibrosis-related markers such as protein kinase C, protein kinase CβII and TGFβ-1 [94]. Moreover, Wang investigated the mechanism by which genistein induces autophagy in renal podocytes, which has been confirmed to be essential for the progression of DN. The results suggested that 20 μM genistein effectively reversed the expression of autophagy-related proteins downregulated by MyD88 siRNA, which indicated that genistein could be a promising agent for treating DN by promoting autophagy in mouse podocyte cells [95].

### Proanthocyanidins

Proanthocyanidin (Fig. 2i) is a key bioflavonoid mainly derived from grape seed and French maritime pine bark that has been found to effectively remove free radicals in the human body as a natural antioxidant [96, 97]. In recent years, proanthocyanidin has been widely investigated as a therapeutic agent to protect diabetic kidneys through antioxidant actions.

Ding found that proanthocyanidin at a dose of 250 mg/kg activates the Nrf2 signalling pathway and increases the levels of superoxide dismutase, increases the total antioxidative capability, increases glutathione and upregulates the expression levels of Nrf2, HO-1, glutathione S-transferase, and NADH:quinone oxidoreductase, resulting in the reversal of renal damage in streptozotocin-induced DN rats [98]. In addition, Gao and colleagues found that 250 mg/kg proanthocyanidin effectively protected renal function and attenuated endoplasmic reticulum stress-induced apoptosis through the Caspase-12 pathway in streptozotocin-induced DN rats. Specifically, the number of TUNEL-positive cells was reduced, and there was an obvious reduction in the protein expression of GRP78, p-ERK, and Caspase-12 [99]. In the same model, Li demonstrated that treatment with proanthocyanidin at a dose of 250 mg/kg strikingly reversed kidney damage in diabetic rats. Meanwhile, receptor for advanced glycation end products (RAGE) expression was decreased and the nephrin expression levels were decreased. Thus, it was suggested that the underlying mechanism of proanthocyanidin action is closely associated with decreased expression of the AGE/RAGE axis and increased expression of nephrin in diabetic rats [100]. Additionally, Bao reported that proanthocyanidin activates peroxisome proliferator-activated receptor-γ coactivator 1α (PPAR-γ) in low-dose streptozotocin and high-carbohydrate/high-fat diet-induced diabetic rats and reduces podocyte injury, which may be related to the inhibition of oxidative stress and reduction in mitochondrial dysfunction in the kidney [101]. Moreover, Li and colleagues administered proanthocyanidin at a dose of 500 mg/kg for 4 weeks and found that GSPE reduced the expression of RAGE and connective tissue growth factor (CTGF) in the kidney, which can help reverse the accumulation of extracellular matrix in diabetes, inhibiting the progression of DN [102].

### Other flavonoids

Eriodictyol (Fig. 2m) is a polyphenol flavonoid compound widely distributed in fruits and vegetables, especially in lemons and peanuts, that exerts several pharmacological actions, such as antioxidant, anti-inflammatory, and analgesic activities, as well as improving diabetes and diabetes-related complications [103]. Bai investigated the mechanism by which eriodictyol exerted a therapeutic role on mercury-induced mesangial cells (MCs) in vivo. The results suggested that eriodictyol at a dose of 0 ~ 25 μm protects MCs from mercury stimulation by inhibiting the activity of the Akt/NF-κB pathway, resulting in suppression of the production of extracellular matrix proteins and the secretion of inflammatory cytokines [104].

Morin (Fig. 2n) is a natural bioflavonoid that can be extracted from the bark of *Moraceae* and many Chinese herbal medicines; morin exerts antioxidant, anti-pain, antibacterial, anti-inflammatory, anti-atherosclerotic, blood sugar-lowering and anti-stress effects [105, 106]. Ke and colleagues reported that 25–50 μm morin inhibited the proliferation of rat glomerular mesangial cells and the aggregation of fibronectin induced by high glucose by suppressing the activation of the p38 MAPK and JNK signalling pathways. Therefore, they suggested that morin could be well-suited for the prevention and treatment of diabetic nephropathy [107].

Tangeretin can be extracted from orange peel (Fig. 2o) and is a natural flavonoid with antifungal and antioxidant effects [108]. Kang orally administered 10 mg/kg tangeretin to db/db mice and found that it can reduce glucose oxidative stress-induced and hypoxia-induced podocyte damage and fibrosis in rats by blocking epithelial-to-mesenchymal transition (EMT). The results showed that the expression level of epithelial markers E-cadherin and P-cadherin were decreased and the expression of podocyte slit diaphragm protein was increased [109]. Using the same model, Chen and colleagues found that 0 ~ 25 μm hesperetin significantly inhibited high glucose-induced cell proliferation, oxidative stress, and the expression of extracellular matrix in glomerular MCs, reducing the level of reactive oxygen species (ROS) and malondialdehyde (MDA) and inhibiting the expression of fibronectin (FN) and type IV collagen. Therefore, they suggested that the underlying mechanisms might be related to the ERK signalling pathway [110].

## Discussion and prospects

With the continuous advancement of high-throughput screening technology, active natural products from plants with special chemical structures that exert multiple pharmacological effects have become an indispensable source of new drugs [111]. Flavonoids are abundant in nature. Flavonoids are divided into ten categories according to their chemical structure, six of which are which (flavonoids, flavanones, anthocyanins, flavonols, isoflavones and catechins) are commonly found in the human diet. Bioflavonoids exert a wide range of pharmacological activities, such as antioxidation, anti-inflammatory, antitumour and metabolic regulation activities, and are used for the treatment of various diseases [112]. Interestingly, flavonoids have obvious advantages for the treatment of chronic metabolic diseases. The aim of this narrative review was to summarize the pharmacological activities of flavonoids in DN.

### Key findings

Current research shows that the pathogenesis of DN is highly related to oxidative stress and inflammation. In addition, hyperglycaemia, excessive reactive oxygen species, and renal arterial hypertension caused by impaired podocyte autophagy have also been confirmed to be important causes of DN.

**Fig. 3:**
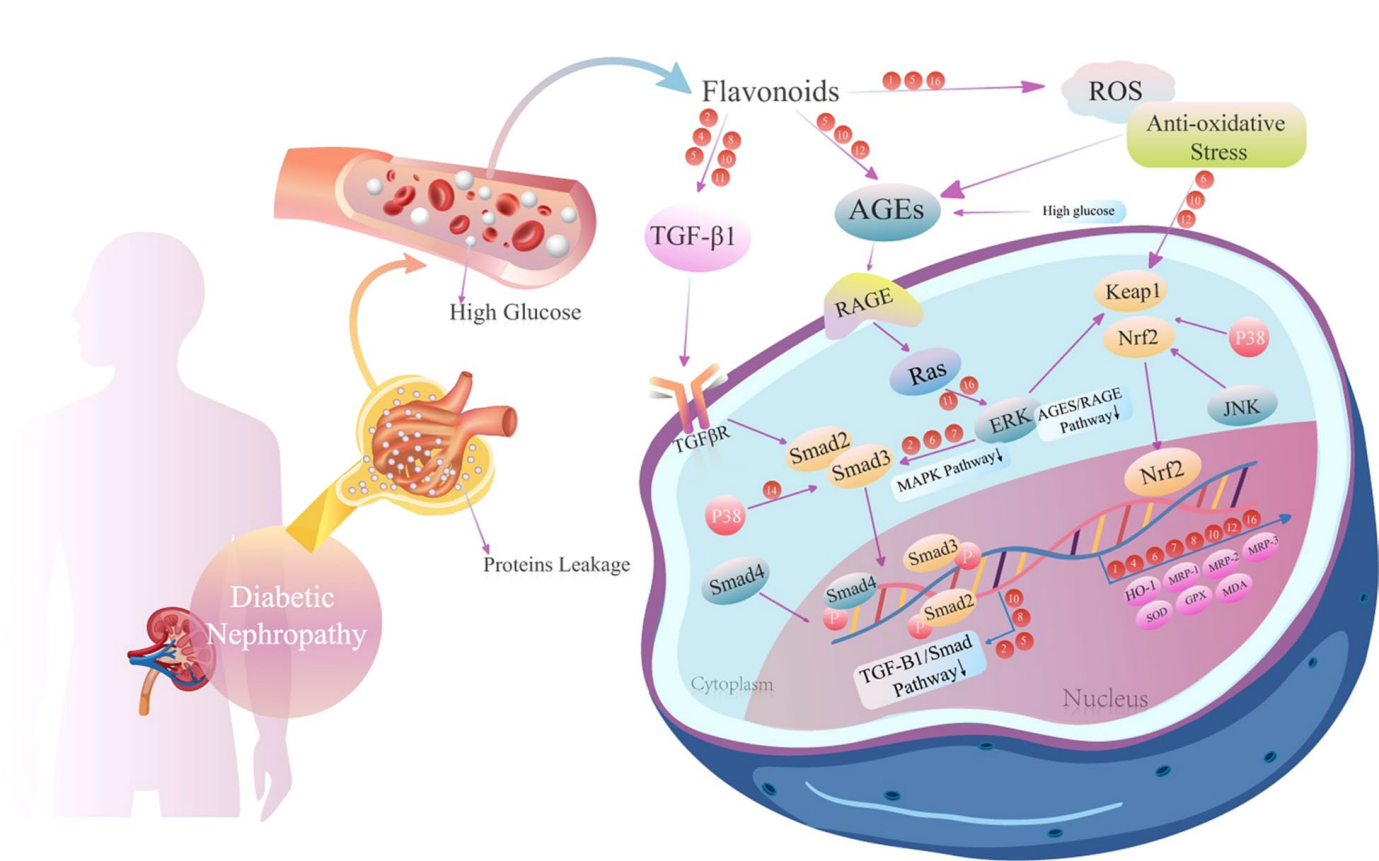
Antioxidant capacities of flavonoids in diabetic nephropathy. The potential mechanisms involved are primarily the downregulation of Nrf2 signalling, MAPK signalling, TGF-β1/Smad signalling and AGES/RAGE signalling. The red circles represent different bioflavonoids in the relevant regulatory pathways (1) quercetin, (2) baicalin, (3) kaempferol, (4) myricetin, (5) rutin, (6) apigenin, (7) luteolin, (8) naringenin, (9) naringin, (10) hesperetin, (11) genistein, (12) proanthocyanidin, (13) eriodictyol, (14) morin, (15) tangeretin

We have summarized 15 common flavonoids. The results show that flavonoids can regulate DN in several ways, including exerting anti-oxidative stress and anti-inflammatory effects. Regarding the oxidative stress pathway, several oxidative stress targets, such as Nrf-2/GSH, ROS production, HO-1, TGF-β1 and AGEs/RAGE, are considered to be key regulatory factors. Notably, most flavonoids can prevent the process of renal fibrosis and alleviate DN by inhibiting the synthesis of TGF-β and ROS; in particular, quercetin, apigenin, baicalin, luteolin, hesperidin, genistein, proanthocyanidin and eriodictyol (Fig. 3).

**Fig. 4:**
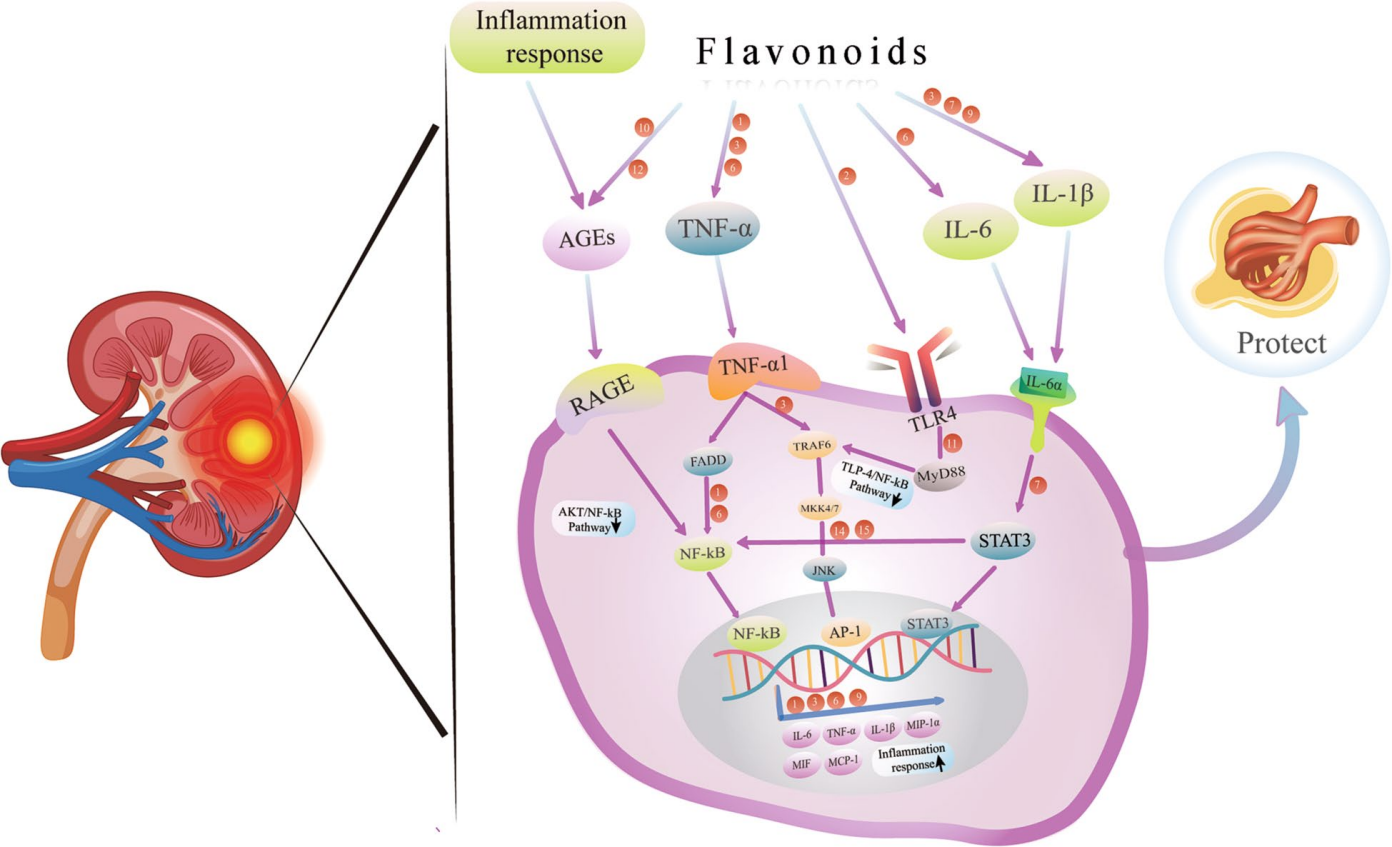
Inflammatory regulation of flavonoids in diabetic nephropathy. The potential mechanisms involved are primarily the downregulation of NF-κB signals, TLR4/NF-κB signals, and AKT/NF-κB signals. The red circles represent different bioflavonoids in the relevant regulatory functions. (1) quercetin, (2) baicalin, (3) kaempferol, (4) myricetin, (5) rutin, (6) apigenin, (7) luteolin, (8) naringenin, (9) naringin, (10) hesperetin, (11) genistein, (12) proanthocyanidin, (13) eriodictyol, (14) morin, (15) tangeretin

In regard to anti-inflammatory effects, proinflammatory factors such as IL-1, IL-6β, TNF-α, SIRT1, NF-κB, and TGF-β1/Smad are thought to be essential for the regulation of inflammation. Quercetin, kaempferol, myricetin, rutin, genistein, proanthocyanidin and eriodictyol have been confirmed to act on the aforementioned targets (Fig. 4).

Furthermore, flavonoids promote podocyte autophagy and inhibit the overactivity of RAAS by suppressing upstream oxidative stress and inflammatory pathways and ultimately alleviate DN. VEGF, ET-1, TGF-β, the P38-MAPK pathway and mTOR are correlated with this regulation mechanism.

Overall, flavonoids reverse the process of renal fibrosis by inhibiting oxidative stress and inflammation and reduce renal cell damage by promoting renal podocyte autophagy.

### Limitations

At present, although several experimental studies have been performed on the pharmacological effects of flavonoids, few clinical studies have been conducted, which indicates that the clinical application of flavonoids is unlikely to occur soon. In addition, this review also has several shortcomings. For example, different DN models may have an impact on the therapeutic effects of flavonoids. In addition, the clinical dosage, dosage formulation, administration method and other key factors were also different among the analyzed studies. Therefore, before clinical trials can be conducted, natural products need to be thoroughly tested in different DN models to determine the most suitable model.

### Prospects and directions

Low bioavailability limits the clinical application of flavonoids. Therefore, new formulations or structural modifications are needed to improve the pharmacokinetic parameters and promote the clinical application of flavonoids [113]. A new viewpoint has recently been put forward indicating that site-specific modification of flavonoids through methylation and/or glycosylation (the process of endogenous occurrence in plants) can be used to improve and adjust their biophysical and pharmacokinetic characteristics [114].

On the other hand, flavonoids can be used as lead compounds for the development of therapeutic drugs for DN. Similar structural modifications were made to artemisinin to generate dihydroartemisinin, which has more powerful antimalarial effects [115]. In addition, the synergistic effect and mechanism of the combination of flavonoids and other ingredients should be considered. For instance, the realgar natural indigo tablet, a combination of arsenic trioxide and tanshinone, has a therapeutic effect on acute promyelocytic leukaemia [116]. Many studies have demonstrated that natural ingredients typically act on multiple targets instead of a single target [117]. In addition, many diseases are complex network signal pathway disorders. Therefore, combination therapy using two or more flavonoids might be a possible method to prevent and treat DN [118]. As network pharmacology and metabolomics technologies have been improved in recent years, the pharmaceuticalization of flavonoid natural ingredients has greater potential [119, 120]. In short, flavonoids are promising drugs for the treatment of DN.

## Conclusion

In summary, bioflavonoids play a multi-target and multi-pathway role in DN therapy, especially via anti-oxidative stress and anti-inflammatory effects that correlate with apoptosis, glomerular protection and kidney fibrosis. In addition, the protective effects of bioflavonoids against DN indicate the potential of flavonoids for DN treatment, and as relevant clinical studies are lacking, further research is warranted in this area.

## Data Availability

All data are available in the manuscript and they are showed in figures and tables.

## Abbreviations

DN: Diabetic nephropathy
Ang II: Angiotensin II
ET-1: Endothelin 1
DKD: Kidney disease
SOD: Superoxide dismutase
CAT: Catalase
ROS: Oxygen species
CTGF: Growth factor
UAE: Urine albumin excretion
sCr: Serum creatinine
BUN: Blood urea nitrogen
Ccr: Creatinine clearance
db/db: Leprdb/Leprdb
LDLr: Low-density lipoprotein
ICAM-1: Intercellular adhesion molecular-1
HG: High glucose
HMC: Human mesangial cell
MAPK: Mitogen-activated protein kinase
IGF: Insulin-like growth factor
TRAF6: Tumor necrosis factor receptor associated factor 6
GPx: Glutathione peroxidase
XO: Xanthine oxidase
GLUT-2: Glucose transporter-2
GLUT-4: Glucose transporter-4
IRS-1: Insulin receptor-1
IRS-2: Insulin receptor-2
PKB: Protein kinase B
TGF-β1: Transforming growth factor-β1
VEGF: Vascular endothelial growth factor
MMPs: Matrix metalloproteinases
Nrf2: NF-E2-related factor 2
HO-1: Heme oxygenase-1
HK-2: Human renal tubular epithelial cell line
SLNPs: Solid lipid nanoparticle
IL-1β: Interleukin-1β
GSPE: Grape seed proantho-cyanidin extracts
MDA: Malondialdehyde
IL-1: Inflammatory factor
Glo-1: Glyoxalase 1
ILK: Integrin-linked kinase
MCP-1: Monocyte chemoattractant protein-1
PPAR-γ: Peroxisome proliferator-activated receptor-γ coactivator 1α
MCs: Mesangial cells
FN: Fibronectin
EMT: Epithelial-to-mesenchymal transition
ACEI: Angiotensin-converting enzyme inhibitors
ARB: Angiotensin receptor antagonists
UAER: Urinary albumin excretion rate
FBG: Fasting blood glucose
hs-CRP: High-sensitivity C reactive protein
